# Increased environmental temperature normalizes energy metabolism outputs between normal and Ames dwarf mice

**DOI:** 10.18632/aging.101582

**Published:** 2018-10-18

**Authors:** Justin Darcy, Samuel McFadden, Yimin Fang, Darlene E. Berryman, Edward O. List, Nicholas Milcik, Andrzej Bartke

**Affiliations:** 1 Division of Geriatric Research, Department of Internal Medicine, Southern Illinois University School of Medicine, Springfield, IL 62702, USA; 2 Department of Medical Microbiology, Immunology and Cell Biology, Southern Illinois University School of Medicine, Springfield, IL 62702, USA; 3 Edison Biotechnology Institute, Ohio University, Athens, OH 45701, USA; 4 Department of Biomedical Sciences, Heritage College of Osteopathic Medicine, Ohio University, Athens, OH 45701, USA; 5 The Diabetes Institute at Ohio University, Ohio University, Athens, OH 45701, USA; 6 Current Affiliation: Section on Integrative Physiology and Metabolism, Joslin Diabetes Center, Harvard Medical School, Boston, MA 02115, USA

**Keywords:** Ames dwarf, energy metabolism, thermogenesis, aging

## Abstract

Ames dwarf (*Prop1^df^*) mice possess a loss-of-function mutation that results in deficiency of growth hormone, prolactin, and thyroid-stimulating hormone, as well as exceptional longevity. Work in other laboratories suggests that increased respiration and lipid utilization are important for maximizing mammalian longevity. Interestingly, these phenotypes are observed in Ames dwarf mice. We recently demonstrated that Ames dwarf mice have hyperactive brown adipose tissue (BAT), and hypothesized that this may in part be due to their increased surface to mass ratio leading to increased heat loss and an increased demand for thermogenesis. Here, we used increased environmental temperature (eT) to interrogate this hypothesis. We found that increased eT diminished BAT activity in Ames dwarf mice, and led to the normalization of both VO_2_ and respiratory quotient between dwarf and normal mice, as well as partial normalization (i.e. impairment) of glucose homeostasis in Ames dwarf mice housed at an increased eT. Together, these data suggest that an increased demand for thermogenesis is partially responsible for the improved energy metabolism and glucose homeostasis which are observed in Ames dwarf mice.

## Introduction

Ames dwarf mice are diminutive, long-lived mutant mice. Both their size and longevity stem from a recessive *Prophet of Pituitary Factor 1* (*Prop1*) loss-of function mutation [1]. This mutation results in hypopituitarism marked by deficiency of growth hormone (GH), thyroid-stimulating hormone (TSH), and prolactin [2,3]. Downstream endocrine effects of this mutation include severely reduced levels of circulating insulin-like growth factor 1 (IGF-1), triiodothyronine (T_3_), and thyroxine (T_4_) [2]. Beyond living up to 60% longer than their normal littermates (depending on sex and diet), Ames dwarf mice have a remarkable extension of healthspan as demonstrated through their improved cognition [4,5], glucose homeostasis [6,7], and energy metabolism [8].

Contrary to our laboratory’s initial hypothesis that Ames dwarf mice have a slower metabolic rate, and therefore extended longevity (falling in-line with the “Rate of Living Hypothesis” [9]), we found the opposite to be true. Ames dwarf mice have an increased rate of oxygen consumption (VO_2_) per gram of body weight, and they have a reduced respiratory quotient (RQ) implying preferential usage of lipids as an energy source [8]. Since this initial finding, our laboratory has demonstrated the relationship between decreased somatotropic (GH and IGF-1) signaling and improved energy metabolism in several studies [10–13]. This relationship appears counterintuitive given i) GH is a highly lipolytic hormone, so its absence should theoretically lower the usage of lipids as metabolic fuel, ii) the severe hypopituitarism of dwarf mice should presumably lead to a slower rate of respiration, and iii) Ames dwarf mice have decreased levels of reactive oxygen species (ROS) [14–16], which given their increased metabolic rate, is opposite to the Rate of Living Hypothesis. This conundrum has been somewhat resolved by the reported benefits of uncoupling the mitochondrial electron transport chain [17]. The “Uncoupling to Survive Hypothesis” was first proposed by Brand [17], and described a mechanism by which increased metabolic rate alters mitochondrial physiology in a way that promotes less ROS production. This relationship between “improved” energy metabolism and longevity have been demonstrated in Ames dwarf mice, as well as other mouse models [18,19]. Despite this relationship, a mechanism that links the severe endocrine deficits of Ames dwarf mice with their improved energy metabolism is still unclear, though recent findings suggest some possibilities. Presumably, due to an increased thermogenic demand, Ames dwarf mice have hyperactive brown adipose tissue (BAT) [13]. We hypothesize that the increased thermogenic demand and subsequent increased BAT activity may be partially responsible for the improved energy metabolism observed in Ames dwarf mice.

To investigate this relationship, we housed Ames dwarf mice and their normal littermates at room temperature and at an increased environmental temperature (eT) of 30˚C, which corresponds to the reported thermoneutral temperature for genetically normal mice. At this temperature, we observed an increase in adiposity in both genotypes of mice, and an increase in overall body weight in dwarf mice without significant alterations in food consumption. This was accompanied by an increase in subcutaneous adipocyte size and altered BAT structure. Moreover, BAT activity was diminished in Ames dwarf mice, and there was a normalization of both VO_2_ and RQ between the genotypes. Lastly, we observed some impairment (that is, partial normalization) in glucose homeostasis in these animals when placed at an increased eT. Together, these findings illustrate that the thermogenic demand and subsequent activation of BAT may play a role in the energy metabolism and glucose homeostasis of Ames dwarf mice.

## RESULTS

### Increased eT results in dwarf mice gaining weight without altering food consumption

**Figure 1:**
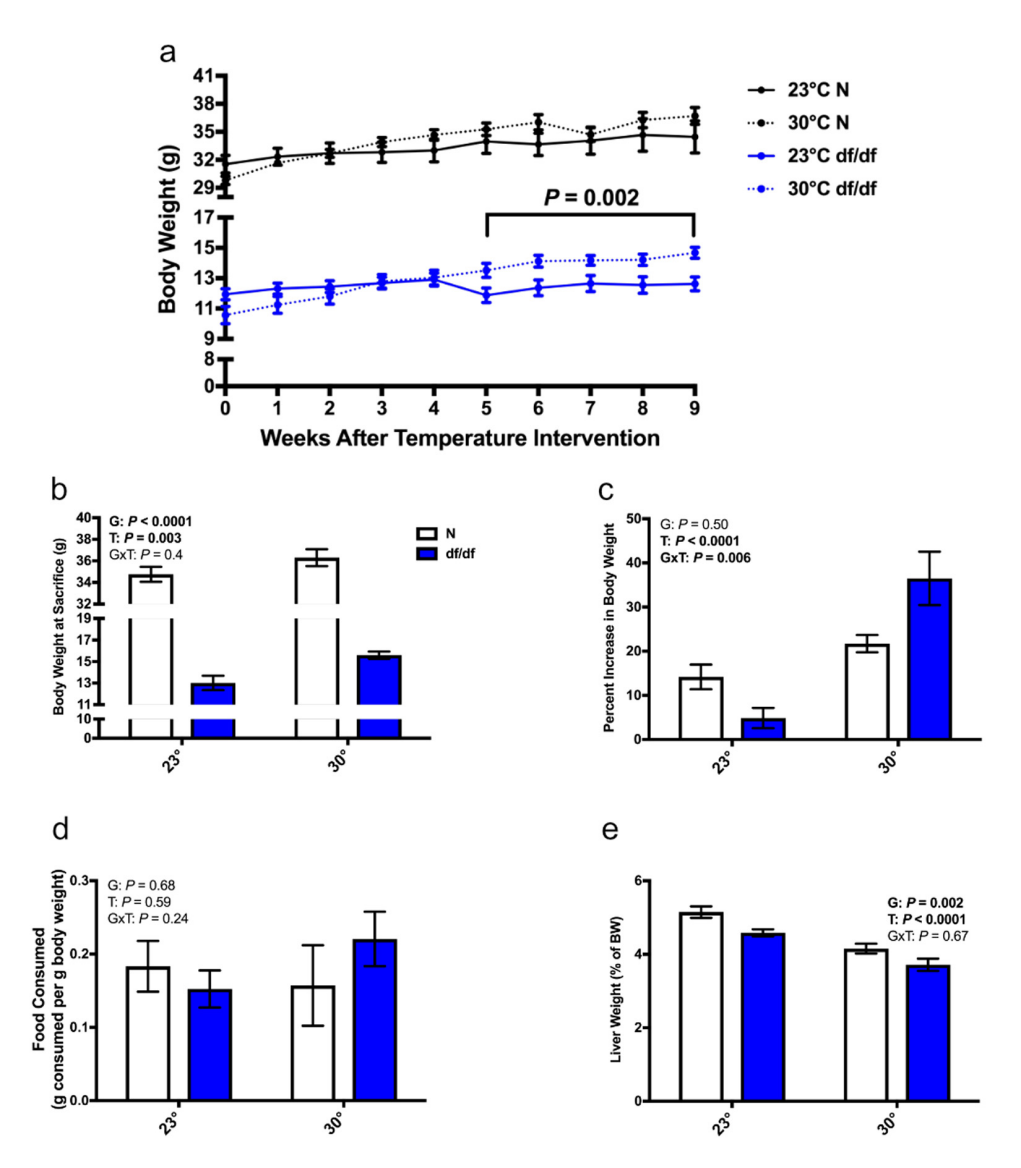
**Increased eT results in dwarf mice gaining weight without altering food consumption.** (**a**) Body weight curve, (**b**) body weight at sacrifice, (**c**) percent increase in body weight from the time of temperature intervention, (**d**) food consumption per g body weight over a 24-hour period, (**e**) liver weight as a % of body weight (n = 6-8 except panel d where n = 4-7). N = normal, df/df = dwarf. The results of the Two-way ANOVA are reported as G (effect of genotype), T (effect of temperature), and GxT (interaction of genotype and temperature). Significant effects (*P* < 0.05) are in bold text. For panel A, a Two-way ANOVA with repeated measures was used to determine significance.

Increased eT lowers the need for thermogenesis, which in turn can lead to alterations in body weight. Interestingly, dwarf mice began to gain body weight 5 weeks after being switched to an increased eT, while this temperature had a less dramatic effect on the body weight of normal mice (Fig. 1a). At the time of sacrifice, there was a significant effect of both genotype and temperature on body weight. Ames dwarf mice at an increased eT weighed approximately 20% more than their room temperature controls, while normal mice at an increased eT weighed approximately 4% more than their room temperature controls (Fig. 1b). This is further illustrated by the percent increase in body weight. Ames dwarf mice gained considerably more weight at an elevated eT than their normal littermates did, which resulted in a significant interaction of genotype and temperature (Fig. 1c). Importantly, the increase in body weight appeared independent of food consumption, as neither genotype significantly differed from their room temperature controls (Fig. 1d and Supp. Fig. 1a). Despite gaining body weight, there was a significant effect of genotype and temperature on liver weight relative to body weight. Normal mice had a larger relative liver weight than their dwarf counterparts, and both genotypes had a smaller relative liver weight at an increased eT than their respective room temperature controls (Fig. 1e). The change in liver weight at an increased eT was only true for normal mice when examining the raw liver weight (Supp. Fig. 1b).

### Increased eT increased adiposity in both normal and dwarf mice

**Figure 2:**
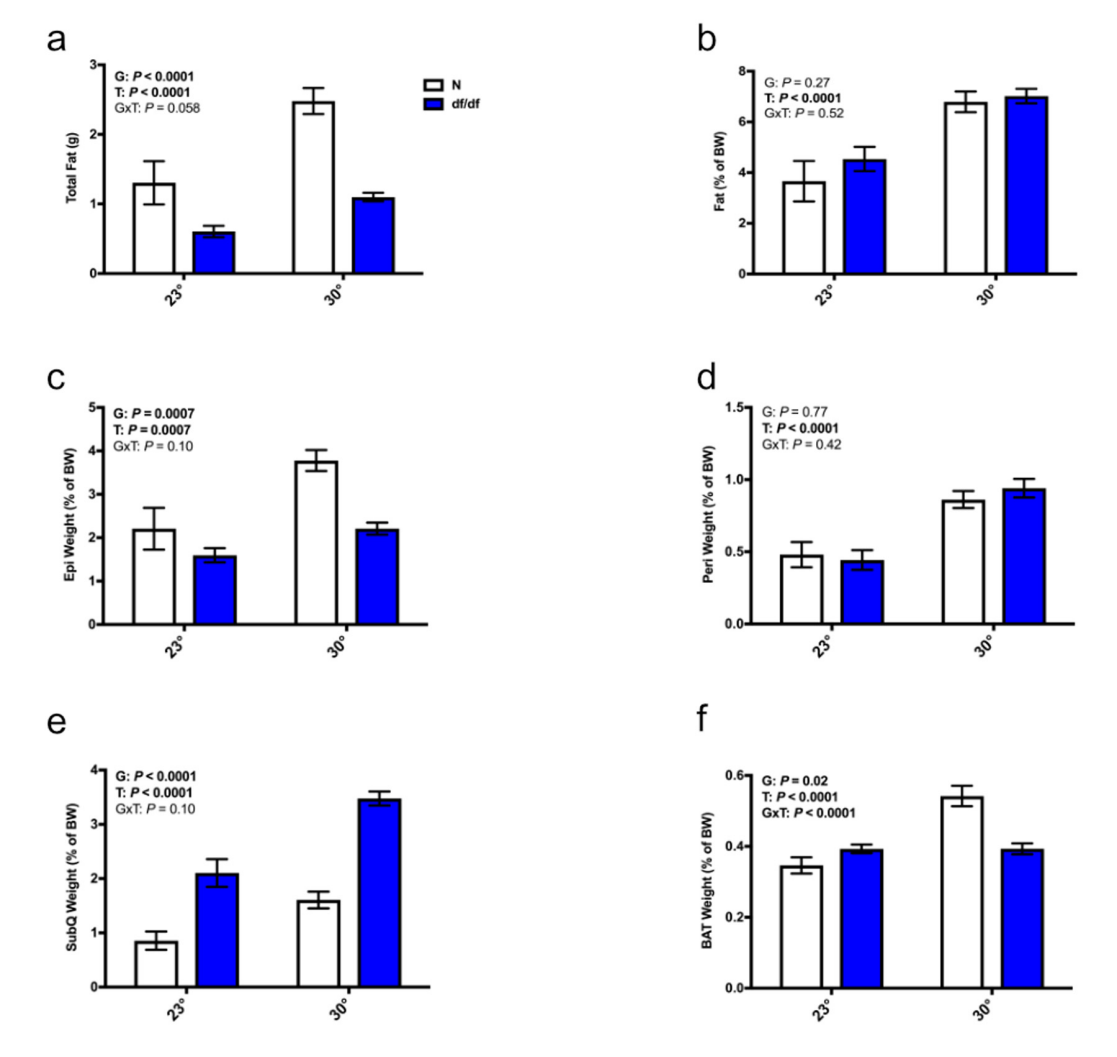
**Increased eT increased adiposity in both normal and dwarf mice.** (**a**) Total fat weight from the epididymal, perirenal, subcutaneous, and brown adipose depots, (**b**) percent body fat, (**c**) weight of the epididymal fat depot as a % body weight, (**d**) weight of the perirenal fat depot as a % body weight, (**e**) weight of the subcutaneous fat depot as a % body weight, (**f**) weight of the brown fat depot as a % body weight (n = 6-8). N = normal, df/df = dwarf. The results of the Two-way ANOVA are reported as G (effect of genotype), T (effect of temperature), and GxT (interaction of genotype and temperature). Significant effects (*P* < 0.05) are in bold text.

There was a significant effect of temperature on both total adiposity and percent body fat in both genotypes of mice, where an elevated eT resulted in increased adiposity (Fig. 2a and 2b). Moreover, there was a significant effect of temperature on the relative (g per g body weight) weights of the epididymal, perirenal, and subcutaneous adipose depots. Both genotypes of mice increased fat depot mass at increased eT (Fig. 2c-2e and Supp Fig. 1c-1e). There was also a significant effect of genotype. Normal mice had more epididymal adipose tissue than their dwarf counterparts, whereas dwarf mice had more subcutaneous adipose tissue than their normal counterparts (Fig. 2c and 2e). Of note, at both environmental temperatures, dwarf mice had the same absolute weight of the subcutaneous fat depot as their normal littermates despite having significantly smaller body weights (Supp. Fig. 1e). Interestingly, there was a significant effect of genotype, temperature, and an interaction between genotype and temperature on the relative (g per g body weight) weight of BAT. Dwarf mice had slightly more BAT at room temperature, and normal mice had a sizable increase in their BAT weight at higher eT (Fig. 2f and Supp. Fig. 1f).

### Increased eT resulted in increased subcutaneous white adipocyte size, and altered structure of brown adipocytes

**Figure 3:**
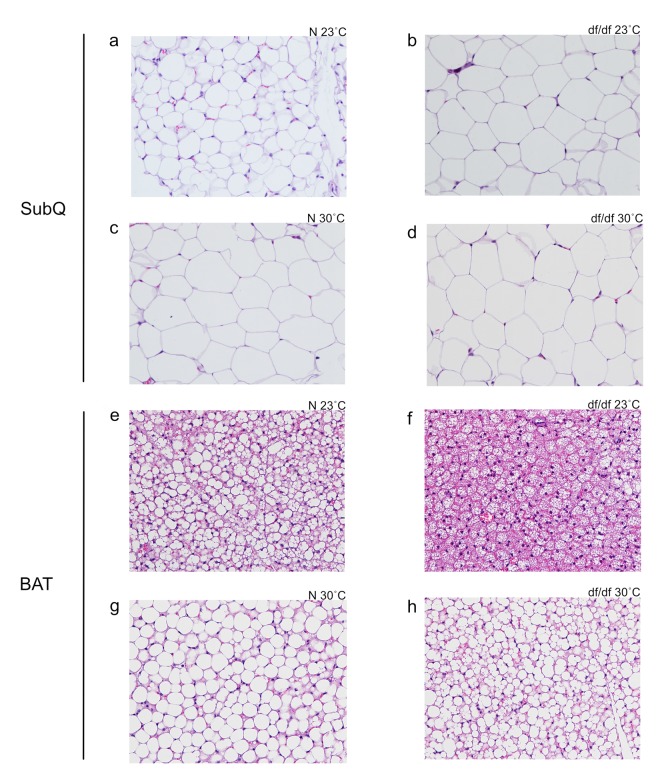
**Increased eT resulted in increased subcutaneous white adipocyte size and altered structure of brown adipocytes.** (**a**) Normal 23˚C subcutaneous, (**b**) dwarf 23˚C subcutaneous, (**c**) normal 30˚C subcutaneous, (**d**) dwarf 30˚C subcutaneous, (**e**) normal 23˚C brown adipose tissue, (**f**) dwarf 23˚C brown adipose tissue, (**g**) normal 30˚C brown adipose tissue, (**h**) dwarf 30˚C brown adipose tissue.

There was a significant effect of both genotype and temperature on subcutaneous adipocyte size (Fig. 3a-3d and Supp. Fig. 2a). Ames dwarf mice had larger adipocytes, and elevated eT increased adipocyte size in both genotypes. Despite changes in the relative tissue weights, there was no evident effect of genotype or temperature on adipocyte size in epididymal or perirenal adipose tissue (data not shown). Brown adipocyte morphology was drastically altered between genotypes and temperatures. As previously reported at room temperature [13], normal mice had a clear accumulation of lipids in their BAT (Fig. 3e-3f). At an elevated eT, both genotypes accumulated lipids, and the apparent difference between the genotypes was diminished (Fig. 3g-3h).

### Increased eT diminished thermogenic gene expression and energy metabolism in dwarf mice

**Figure 4:**
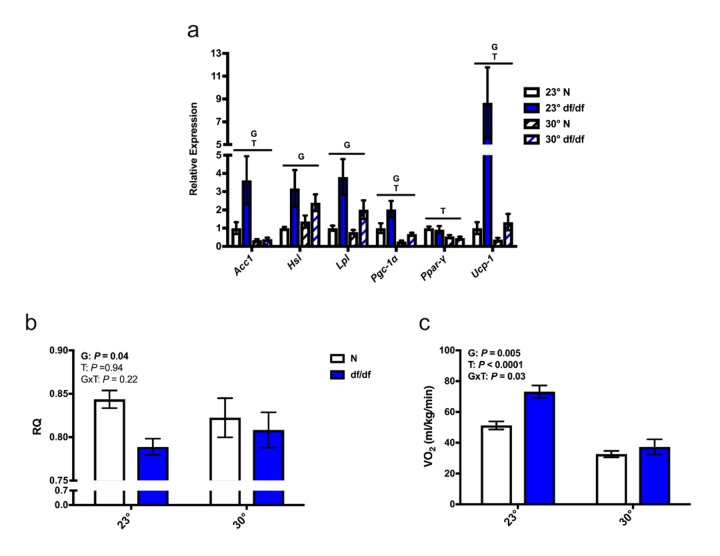
**Increased eT diminished non-shivering thermogenesis and energy metabolism in dwarf mice.** (**a**) Gene expression in brown adipose tissue, (**b**) respiratory quotient, and (**c**) rate of oxygen consumption (n = 5-8). N = normal, df/df = dwarf, RQ = respiratory quotient, VO_2_ = rate of oxygen consumption. The results of the Two-way ANOVA are reported as G (effect of genotype), T (effect of temperature), and GxT (interaction of genotype and temperature). Significant effects (*P* < 0.05) are in bold text.

Due to the alterations in BAT microstructure, we examined the effects of increased eT on thermogenic gene expression. As previously reported [13], we found a significant effect of genotype on the expression of several thermogenic genes in BAT at room temperature (Fig. 4a), which were upregulated in dwarf mice. Moreover, the mRNA levels of *Acc1*, *Pgc-1α*, *Ppar-γ*, and *Ucp-1* were all downregulated by an increase in eT in both genotypes of mice, while mRNA levels of hormone-sensitive lipase (*Hsl*) and lipoprotein lipase (*Lpl*) showed a numerical trend towards diminished expression in dwarf mice. Along with the decreased expression of thermogenic genes, we observed alterations in some parameters of energy metabolism. There was a significant effect of genotype on RQ, and a “normalization” of RQ between the two genotypes at an elevated eT (Post-hoc *P* = 0.92) (Fig. 4b). Moreover, there was a significant effect of genotype, temperature, and interaction between genotype and temperature on VO_2_, which was also normalized between the two genotypes at an increased eT (Fig. 4c).

### Increased eT perturbed glucose homeostasis in both normal and dwarf mice

**Figure 5:**
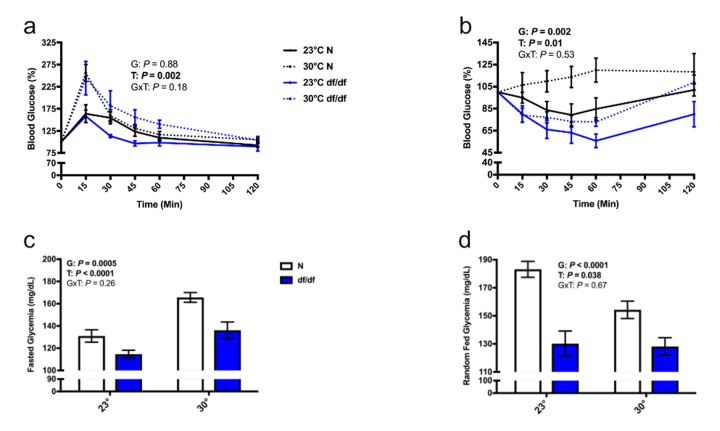
**Increased eT perturbed glucose homeostasis in both normal and dwarf mice.** (**a**) Glucose tolerance test, (**b**) insulin tolerance test, (**c**) fasted blood glucose, and (**d**) fed blood glucose levels (n = 5-9). N = normal, df/df = dwarf. The results of the Two-way ANOVA are reported as G (effect of genotype), T (effect of temperature), and GxT (interaction of genotype and temperature). Significant effects (*P* < 0.05) are in bold text.

While there was no significant effect of genotype on glucose tolerance (Fig. 5a and Supp. Fig. 3a), there was a significant effect of genotype on insulin sensitivity (Fig. 5b and Supp. Fig. 3b), where dwarf mice are more insulin sensitive. Importantly, there was a significant effect of temperature on both glucose tolerance and insulin sensitivity, where both normal and dwarf mice became more glucose intolerant and insulin resistant at an increased eT. Moreover, there was a significant effect of temperature and genotype on fasted blood glucose levels, where dwarf mice had lower blood glucose compared to their normal counterparts at both temperatures, and both genotypes of mice had elevated blood glucose levels at an elevated eT (Fig. 5c). There was also a significant effect of genotype and temperature on fed blood glucose, where dwarf mice had lower fed glucose than their normal counterparts at both temperatures. However, in contrast to the increase in fasted glucose at an elevated eT, both genotypes had lower fed glucose than their respective room temperature controls (Fig. 5d).

## DISCUSSION

Currently, comorbidities as a result of aging and obesity place tremendous stress on healthcare systems. Energy metabolism (including BAT-thermogenesis) is therefore a critical area to examine since it directly impacts both aging and obesity. This relationship can be seen through a recent study by Oliverio et al. that utilized BAT from Ames dwarf, *Ercc1-/-* (premature aging) and diet-induced obesity (DIO) mice to demonstrate that alterations in BAT microRNA profiles in *Ercc1-/-* and DIO mice are opposite to those of Ames dwarf mice [20]. While this study and others have demonstrated differences in BAT function between Ames dwarf mice and their normal littermates, little in the way of mechanism has been evaluated. We previously hypothesized that the increased demand for thermogenesis in these diminutive animals drives their enhanced BAT function, and therefore, their altered energy metabolism. To this end, we housed Ames dwarf mice and their normal littermates at standard room temperature and at an increased eT (thermoneutrality for genetically normal mice, 30˚C) to minimize their thermogenic demand.

We were surprised to see that when exposed to an increased eT, Ames dwarf mice had a dramatic percent increase in body weight compared to their room temperature controls, while normal mice had a marginal increase, suggesting there is a genotype difference in either food consumption or energy expenditure. However, neither genotype saw a significant difference in food consumption compared to their respective room temperature controls, leading us to hypothesize that the difference in dwarf body weight at varying temperatures was the result of altered energy metabolism. More on this is discussed later in this section.

To determine if adiposity accounted for the increased body weight, we measured the total weight of adipose tissue, and observed an increase in both genotypes housed at an elevated eT. Moreover, we observed an increase in percent body fat in both genotypes compared to their respective room temperature controls. We did not observe a difference in percent body fat between genotypes at either temperature, however, these mice were relatively young and the “chubby” phenotype observed in Ames dwarf mice is age-dependent [21]. The relative weights of the subcutaneous, perirenal, and epididymal depots all increased based on increased temperature. Moreover, dwarf mice had an increase in relative subcutaneous adipose tissue weight compared to their normal littermates, whereas normal mice had an increase in relative epididymal adipose tissue weight compared to dwarf mice. While the differences in deposition, structure, and function of adipose tissue in GH mutant mice are beyond the scope of this article, interested readers are directed to several relevant review articles [22–24]. Ames dwarfs had an increase in the relative weight of their BAT as previously reported [13]. At an increased eT, normal mice gained considerable weight in their BAT depot while dwarf mice did not. We suspected this may be due to differential lipid accumulation in the adipose depot, however, histological staining did not support this hypothesis. Future work needs to be conducted to explore the source of this difference within the BAT depot as we do not currently have an explanation for this phenomenon.

Due to alterations in adiposity, we examined differences in adipocyte size. To our surprise, only adipocytes from the subcutaneous depot had altered size. What was most interesting, however, was the histological appearance of BAT. As we have previously reported, the normal littermates of Ames dwarf mice have a much greater accumulation of lipids in their BAT at room temperature, and the BAT from dwarf mice is more eosinophilic, both of which indicate that the BAT in dwarf mice may be more active [13]. For reasons we are unsure of, the histological examination of the BAT from the normal littermates at room temperature revealed an unusually large accumulation of lipids. Interestingly, another long-lived GH mutant, the growth hormone receptor knockout (GHR-KO), has a similar distribution of lipid accumulation in their BAT (Bartke and Berryman, unpublished data). Further investigations will need to be conducted to elucidate why this alteration occurs. At an increased eT, both genotypes accumulated lipids in their BAT, and the difference between the adipose tissue structure of dwarf mice and normal mice was nearly eliminated.

Due to the normalization of dwarf and normal brown adipocyte structure at an elevated eT, we suspected thermogenesis may also be impacted. To that end, we examined the expression of several genes that are related to thermogenesis. Except for *Ppar-γ*, all of the thermogenic genes examined were increased in dwarf mice at room temperature. This difference was diminished or eliminated at an elevated eT for every gene except for *Hsl* and *Lpl*, which showed a numerical trend towards normalization. As mentioned earlier in this section, dwarf mice gained both weight and adiposity at an increased eT without altering food consumption, which was likely a result of altered energy metabolism. Moreover, we observed normalization of lipid accumulation in brown adipocytes, as well as thermogenic gene expression in BAT, which further indicates alterations in energy metabolism at an elevated eT. As previously reported [8], we observed a decreased RQ and an increased VO_2_ in dwarf mice at room temperature. Although there was not a significant effect of temperature on RQ, there was a numerical trend towards normalization. Importantly, the large difference in VO_2_ between dwarf and normal mice disappeared at an increased eT. More on this will be discussed later in this section. We acknowledge that the RT-PCR and indirect calorimetry measurements are indirect indexes of thermogenesis.

We next examined glucose tolerance and insulin sensitivity since both BAT [25] and eT [26] have been shown to play a role in glucose homeostasis. Although dwarf mice did clear glucose faster than their normal littermates at the 30- and 45-minute time points, we did not observe any overall statistically significant difference in glucose tolerance between the genotypes at room temperature. However, Ames dwarf mice were significantly more insulin sensitive than their normal littermates at room temperature. Both genotypes exhibited glucose intolerance and insulin resistance at an elevated eT, which was accompanied by an increase in fasted blood glucose. The changes in glucose tolerance and insulin sensitivity were based on injecting glucose and insulin per g body weight, and may be different these were injected at a set amount per animal. Interestingly, we observed a decrease in fed blood glucose levels in both genotypes at an increased eT. Since we believe insulin sensitivity to be a key mechanism by which Ames dwarf mice are long-lived [2] future work involving eT and insulin signaling is of extreme interest.

Aligning with the Uncoupling to Survive Hypothesis, Ames dwarf mice have an increase in their rate of oxygen consumption. This finding appears to be counterintuitive given the hypopituitarism and presumed increased heat loss of these animals. After previously illustrating that Ames dwarf mice have hyperactive BAT, we proposed a hypothesis for their improved energy expenditure: the increased demand for thermogenesis as a result of hypothyroidism and presumable increased heat loss drives increased non-shivering thermogenesis, which in turn, increases energy metabolism. To interrogate this hypothesis, we used elevated eT as an intervention. Presumably, at an elevated eT, the demand for thermogenesis should be mitigated. Under this condition, both thermogenic gene expression and VO_2_ were normalized between dwarf and normal mice, supporting our hypothesis. Arguably, the increased demand for thermogenesis overrides the possibly negative impact of hypothyroidism on mitochondrial biogenesis leading to increased uncoupling and beta oxidation of fatty acids. Untangling which aspects of dwarf physiology are largely responsible for this phenomenon is of particular interest for future work.

## MATERIALS AND METHODS

### Animals

Male Ames dwarf mice and their normal littermates were produced in our closed breeding colony at Southern Illinois University School of Medicine (SIUSOM) on a heterogeneous background. Our colony is maintained by mating heterozygous females with homozygous dwarf males while avoiding brother x sister mating. At approximately 4 months-of-age, normal and dwarf mice were housed at room temperature (22-23˚C), or were switched to increased eT (28-30˚C). This age was chosen to closely resemble work previously done on BAT in these animals [13]. All of the mice in this study were maintained on a normal 12hr light:12h dark schedule, and were provided ad libitum access to water and food (LabDiet 5001, with 56% calories from carbohydrates, 29% calories from protein, and 13% calories from fat). Body weight was measured weekly, and all *in vivo* experiments were conducted between 7 and 11 weeks following temperature intervention. All animal protocols for this study were approved by the SIUSOM Animal Care and Use Committee.

### Indirect calorimetry

Indirect calorimetry was performed as previously described [8] using the PhysioScan Metabolic System. In this system, mice are housed individually in metabolic chambers with ad libitum access to food and water. After a 24-hour acclimation period, VO_2_ and RQ measurements were collected every 10 minutes, for 24 hours. Food consumption was also monitored during this period.

### Glucose and insulin tolerance tests

Glucose and insulin tolerance tests were performed as previously described [11,13]. For the glucose tolerance test (GTT), mice were fasted overnight for 16 hours before blood glucose from the tail vein was measured using a glucometer (time 0). After injecting mice i.p. with 2 g of glucose per kg of body weight, sequential blood glucose measurements were taken at 15, 30, 45, 60, and 120 minutes. For the insulin tolerance test (ITT), blood glucose from the tail vein was measured in non-fasted animals. After injecting mice i.p. with 1 IU of porcine insulin per kg of body weight, sequential blood glucose measurements were taken as described above.

### Histology

Fixation, sectioning, and hematoxylin and eosin (H&E) staining was performed using a standard laboratory protocol. Tissue was fixed in formalin, washed with alcohol, embedded in paraffin, and sectioned at 5 µm. The resulting sections were examined using light microscopy at 200x magnification, using a Nikon Eclipse E600 microscope equipped with a Spot RT digital camera microscope, and Nikon Elements software was used to capture the images. Quantification of adipocyte size was performed as previously described [27] using 300 adipocytes.

### RT-PCR

Quantative RT-PCR was performed as previously described [12] using the StepOne System and SYBR Green master mix. In short, tissue was homogenized, RNA was extracted using an RNeasy lipid tissue mini kit following the manufacturer’s instructions, and cDNA was made using an iScript cDNA synthesis kit. Amplification was performed over 40 cycles using mRNA-specific primers (Supplementary Table 1). *B2m* was used as a housekeeping gene, and fold change was calculated as previously described [13,28].

### Statistical analysis

Statistical analyses were conducted using a Two-way ANOVA to test for significance of genotype (normal x dwarf), temperature (23˚C x 30˚C), and an interaction between genotype and temperature. For the ITT and GTT, the area under the curve was calculated and used for the Two-way ANOVA. Statistical analyses were conducted using Prism 6 for Mac. All figures are represented as mean ± SEM, with statistical significance set to *P* < 0.05.

## Supplementary Material

Supplementary Figures

Supplementary Table
