# Supplementary material for: Increased environmental temperature normalizes energy metabolism outputs between normal and Ames dwarf mice
Source: Aging (Albany NY). 2018 Oct 18;10(10):2709–22. doi: 10.18632/aging.101582 (PMC6224234; doi:10.18632/aging.101582)
Supplement: Supplementary Figures [file aging-10-101582-s001.pdf]

SUPPLEMENTARY FIGURES

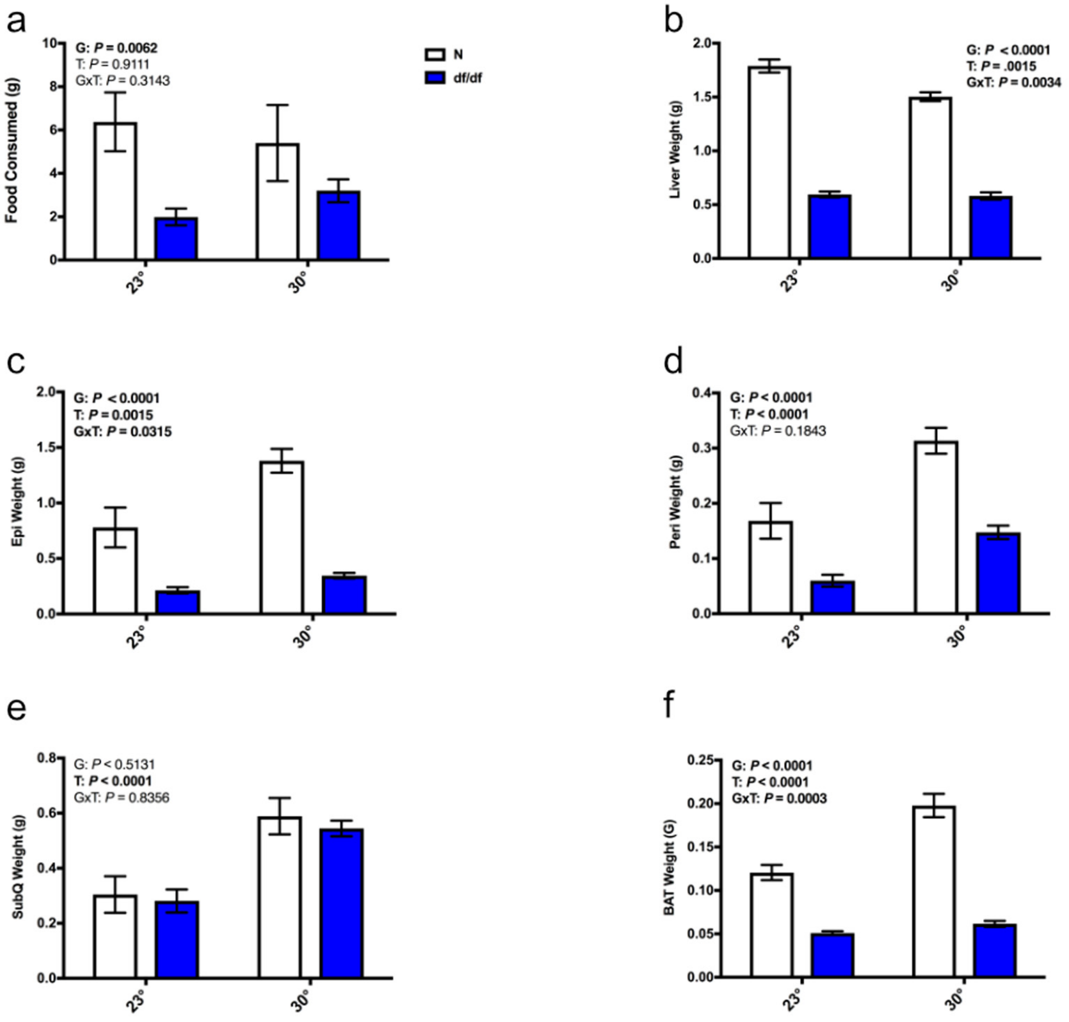

Supplementary Figure 1. Increased eT results in dwarf mice gaining weight without altering food consumption. (**a**) Absolute food consumption over a 24-hour period, (**b**) liver weight, (**c**) epididymal fat depot weight, (**d**) perirenal fat depot weight, (**e**) subcutaneous fat depot weight, and (**f**) brown fat depot weight (n = 6-8 except panel a where n = 4-7). N = normal, df/df = dwarf. The results of the Two-way ANOVA are reported as G (effect of genotype), T (effect of temperature), and GxT (interaction of genotype and temperature). Significant effects ($P < 0.05$) are in bold text.

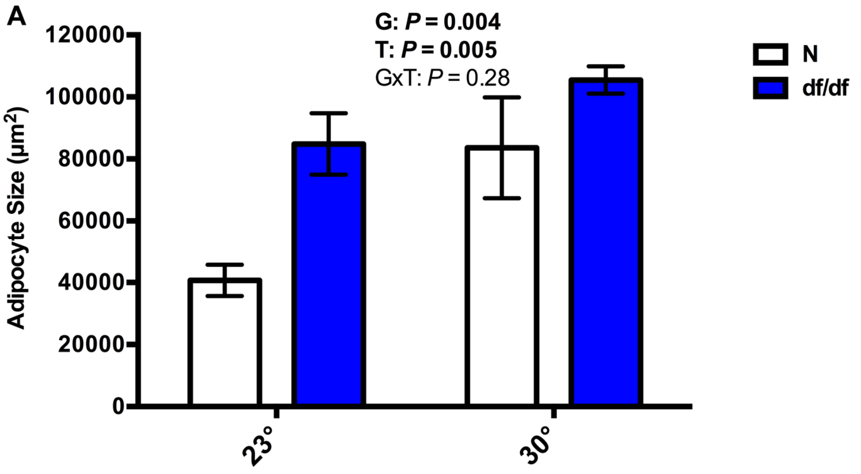

Supplementary Figure 2. Increased eT increases subcutaneous adipocyte size. (**a**) subcutaneous adipocyte size (n = 6). N = normal, df/df = dwarf. The results of the Two-way ANOVA are reported as G (effect of genotype), T (effect of temperature), and GxT (interaction of genotype and temperature). Significant effects (*P* < 0.05) are in bold text.

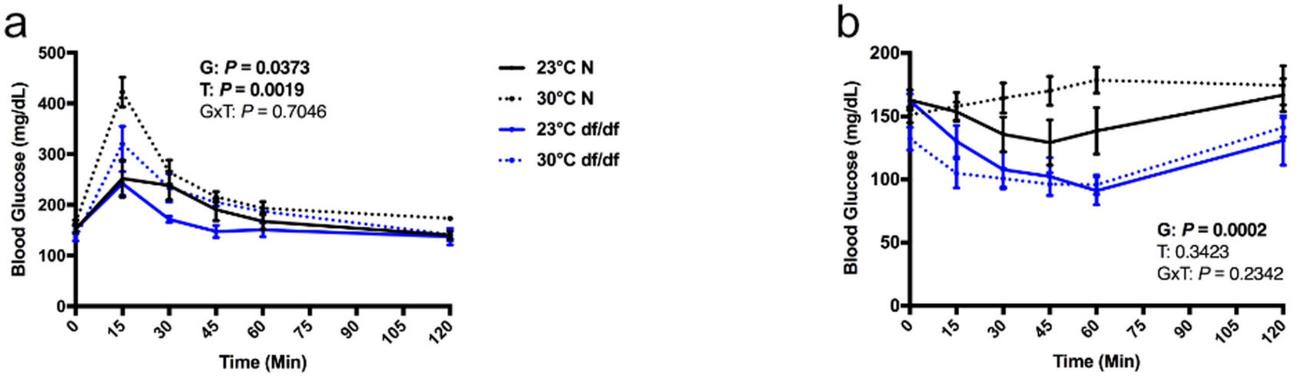

Supplementary Figure 3. Increased eT perturbed glucose homeostasis in both normal and dwarf mice. (**a**) Glucose tolerance test and b) insulin tolerance test (n = 6-8). N = normal, df/df = dwarf. (**b**) The results of the Two-way ANOVA are reported as G (effect of genotype), T (effect of temperature), and GxT (interaction of genotype and temperature). Significant effects (*P* < 0.05) are in bold text.
