# Supplementary material for: Increased environmental temperature normalizes energy metabolism outputs between normal and Ames dwarf mice
Source: Aging (Albany NY). 2018 Oct 18;10(10):2709–22. doi: 10.18632/aging.101582 (PMC6224234; doi:10.18632/aging.101582)
Supplement: Supplementary Table [file aging-10-101582-s002.pdf]

## SUPPLEMENTARY TABLE

**Supplementary Table 1.** Primer Table. mRNA-specific primers used to create cDNA. All primers are listed 5' – 3'.

| Gene | Forward | Reverse |
|---|---|---|
| *Acc1* | GTCCCCAGGGATGAACCAATA | GCCATGCTCAAGGAAAGTAGC |
| *B2m* | AAGTATACTCACGCCACCCA | AAGACCAGTCCTTGCTGAAG |
| *Hsl* | CACCCATAGTCAAGAACCCCTTC | TCTACCACTTTCAGCGTCACCG |
| *Lpl* | AGGGCTCTGCCTGAGTTGTA | AGAAATTTCGAAGGCCTGGT |
| *Pgc-1α* | GACTCAGTGTCACCACCGAAA | TGAACGAGAGCGCATCCTT |
| *Ppar-γ* | ACCCCCTGCTCCAGGAGAT | TGCAATCAATAGAAGGAACACGT |
| *Ucp-1* | AGGCTTCCAGTACCATTAGGT | CTGAGTGAGGCAAAGCTGATTT |
